# Enhancing User Compliance of Wearable Hip Protectors in Long-Term Care through Education and Technology

**DOI:** 10.1093/geroni/igaf122.2626

**Published:** 2025-12-31

**Authors:** Yijian Yang, Xu Song

**Affiliations:** The Chinese University of Hong Kong, Hong Kong, China (People’s Republic); The Chinese University of Hong Kong, Hong Kong, China (People’s Republic)

## Abstract

More than 95% of hip fractures in older adults are caused by falls. In long-term care, the rates of falls and hip fractures are twice as high as those in the community. Although hip protectors (HPs) have been promoted to reduce hip fractures, the user compliance (acceptance and adherence) is generally low. This study aimed to enhance compliance of wearable HPs in long-term care through structured education and innovative design of HPs. 101 care workers from six long-term care facilities attended a 60-minute educational session. 290 residents (age: 79±10) were recruited and 152 accepted to wear HPs. Adherence with wearing HPs was recorded on weekly-basis over 12 months. Generalized linear models were used to examine factors associated with adherence. Meanwhile, we developed a novel HP through 3D-printing. Specifically, selective laser sintering technology was applied to fabricate thermoplastic polyurethane materials, and a triply periodic minimal surface structure-infill scheme was used to generate HPs. Biomechanical performance of the 3D-printed HP was tested using a drop tower. Our results showed a satisfactory adherence rate [48.0% (73/152)] of wearing HPs through education. Participants with dementia or using mobility aids were more likely to wear HPs (Odds Ratio (95% CI): 1.42 (1.10–1.83) and 1.36 (1.05–1.78), respectively). Age, sex, hypertension, osteoporosis, and use of antipsychotic were not associated with adherence (p > 0.05). Moreover, our 3D-printed HP is light (48g) and breathable, with 42% force attenuation. Our findings suggest that user compliance can be enhanced through education and better design of HPs with an increased comfort.

